# Honey Bee Larval Hemolymph as a Source of Key Nutrients and Proteins Offers a Promising Medium for *Varroa destructor* Artificial Rearing

**DOI:** 10.3390/ijms241512443

**Published:** 2023-08-04

**Authors:** Vincent Piou, Caroline Vilarem, Solène Blanchard, Jean-Marc Strub, Fabrice Bertile, Michel Bocquet, Karim Arafah, Philippe Bulet, Angélique Vétillard

**Affiliations:** 1 Laboratoire Evolution et Diversité Biologique, UMR5174, CNRS-Université de Toulouse III-IRD—Université Paul Sabatier, 31077 Toulouse, France; vincent.piou@univ-tlse3.fr (V.P.); solene.blanchard@univ-tlse3.fr (S.B.); 2 M2i Biocontrol–Entreprise SAS, 46140 Parnac, France; 3 Laboratoire de Spectrométrie de Masse Bio-Organique, Département des Sciences Analytiques, Institut Pluridisciplinaire Hubert Curien, UMR 7178 (CNRS-UdS), 67037 Strasbourg, Francefbertile@unistra.fr (F.B.); 4 Apimedia, 74370 Pringy, Annecy, France; 5 Plateforme BioPark d’Archamps, 74160 Archamps, France; 6 Institute pour l’Avancée des Biosciences, CR Université Grenoble Alpes, Inserm U1209, CNRS UMR 5309, 38000 Grenoble, France; 7 Conservatoire National des Arts et Métiers (CNAM), Unité Métabiot, 22440 Ploufragan, France

**Keywords:** mite, artificial dummy, hemolymph, parasite, protein, nutrition, insect physiology

## Abstract

*Varroa destructor*, a major ectoparasite of the Western honey bee *Apis mellifera*, is a widespread pest that damages colonies in the Northern Hemisphere. Throughout their lifecycle, *V. destructor* females feed on almost every developmental stage of their host, from the last larval instar to the adult. The parasite is thought to feed on hemolymph and fat body, although its exact diet and nutritional requirements are poorly known. Using artificial Parafilm™ dummies, we explored the nutrition of *V. destructor* females and assessed their survival when fed on hemolymph from bee larvae, pupae, or adults. We compared the results with mites fed on synthetic solutions or filtered larval hemolymph. The results showed that the parasites could survive for several days or weeks on different diets. Bee larval hemolymph yielded the highest survival rates, and filtered larval plasma was sufficient to maintain the mites for 14 days or more. This cell-free solution therefore theoretically contains all the necessary nutrients for mite survival. Because some bee proteins are known to be hijacked without being digested by the parasite, we decided to run a proteomic analysis of larval honey bee plasma to highlight the most common proteins in our samples. A list of 54 proteins was compiled, including several energy metabolism proteins such as Vitellogenin, Hexamerin, or Transferrins. These molecules represent key nutrient candidates that could be crucial for *V. destructor* survival.

## 1. Introduction

Each organism comes with its load of parasites and pathogens, either as a result of a long coevolution process or because of new contacts between two species [1,2]. These parasites can have deleterious impacts on ecosystems or animal and human health [3,4,5]. Most of the time, parasitic species are studied in the context of a two- or even three-way relationship with their host and with the pathogens they transmit [6,7]. Although challenging, studying such harmful species separately in laboratory conditions represents a complementary and more controlled approach [8,9]. The difficulty in focusing on parasites alone is to isolate them and keep them away from their host. Artificial feeding systems are often necessary to successfully maintain and study parasites separately over prolonged periods [10]. Many protocols exist for ticks or hematophagous insects and have enlightened our understanding of parasite disease transmission and susceptibility to treatments [11,12,13,14]. Since standardized laboratory media and rearing environments can be artificially modified, these methods are also unrivaled for exploring the feeding biology of parasitic pests.

Because of its impact on the Western honey bee (*Apis mellifera*), beekeeping, and agriculture, the ectoparasite *Varroa destructor* is one of the main pests studied throughout the world [15,16,17]. Since it is an obligate parasite, it is only possible to isolate the mite from its host and to maintain it in the laboratory for limited periods. Until now, most rearing methods have relied on easy-to-make Parafilm™ membranes but on complex synthetic feeding solutions [18,19,20]. Recently, the importance of 3D round-shaped substrates in triggering mite feeding behavior under artificial conditions was highlighted [21]. The use of a phosphate-buffered saline (PBS) solution also offers a nutrient-deprived control solution that can be employed as a basic medium to be supplemented in *V. destructor* studies [21]. Although the exact composition of the natural, balanced diet remains unknown, testing solutions prepared from honey bee tissue samples can yield better survival rates [22]. Thus, they could represent a reliable alternative to synthetic solutions for laboratory rearing. Altogether, these artificial feeding protocols offer promising prospects for exploring the physiology, feeding behavior, and nutritional requirements of *V. destructor*.

Unlike many other bee parasites, *V. destructor* infests both adult and immature stages of its host throughout its cycle [16,17]. Female mites have to puncture the cuticle of the larval, pupal, or adult host to get nutrients from hemolymph and fat body [22,23,24,25]. However, we do not know whether the necessary nutrients come mostly from lysed hemocytes or fat body cells or directly from molecules circulating in the hemolymph. The precise nature of the proteins, carbohydrates, vitamins, or lipids essential to the mite’s survival and reproduction has never been determined either. In addition to being a potential source of amino acids necessary for protein synthesis, undigested honey bee proteins could act as key nutrients in the mite’s physiology, as they have been found inside *V. destructor* hemocoel and reproductive organs. The hijacking of such molecules could be of prime importance for parasite physiology [24,26]. Determining the nutritional requirements of *V. destructor*, especially in terms of proteins, is thus crucial to better understanding the parasite and rearing it under artificial conditions.

Following improvements in rearing protocols, we experimentally explored the nutrition and feeding requirements of mites under laboratory conditions. In this study, we focused on hemolymph, as it is usually easier to collect in great quantities compared to fat body. Hemolymph from three different stages of honey bee development was tested to assess its nutritional potential as a natural food source. Larval, pupal, or adult hemolymph was included in 3D rounded Parafilm™ dummies to assess the consumption of food and the survival of mites. The idea was to investigate if the nutritional value of hemolymph could vary throughout honey bee development and impact the mite. To further assess whether essential nutrients came from lysed cells or from molecules circulating in the hemolymph, we tested treated larval hemolymph or synthetic media as feeding solutions. Finally, the protein content of filtered cell-free larval hemolymph—used successfully in our rearing bioassays—was analyzed by off-gel proteomics in order to identify protein candidates that could be key nutrients for *V. destructor*.

## 2. Results

### 2.1. Bioassay 1: Effect of Hemolymph from Different Bee Developmental Stages on V. destructor Survival

**Figure 1:**
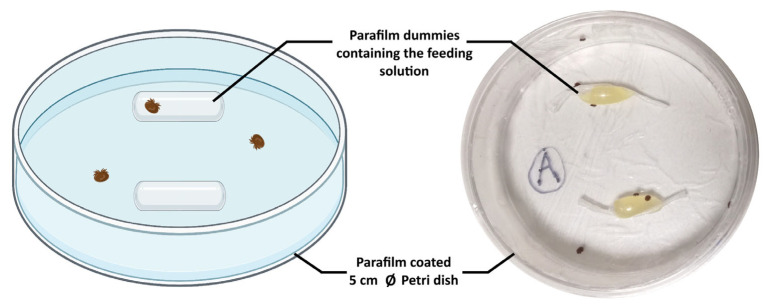
Artificial feeding chambers used to maintain mature female mites. Chambers consist of Petri dishes (Ø 5 cm) covered with Parafilm™. Two dummies containing an artificial feeding solution were placed in the chamber before 10 to 12 females *V. destructor* were inserted.

Since untreated hemolymph can quickly become unsuitable for *V. destructor* feeding after a few days at 34 °C (Figure S1), freshly collected larval, pupal, or adult hemolymph was heated at 65 °C for 7 min (as in [20]). This process prevents contamination and limits clotting or melanization once hemolymph is included into feeding Parafilm™ dummies (displayed in Figure 1).

**Figure 2:**
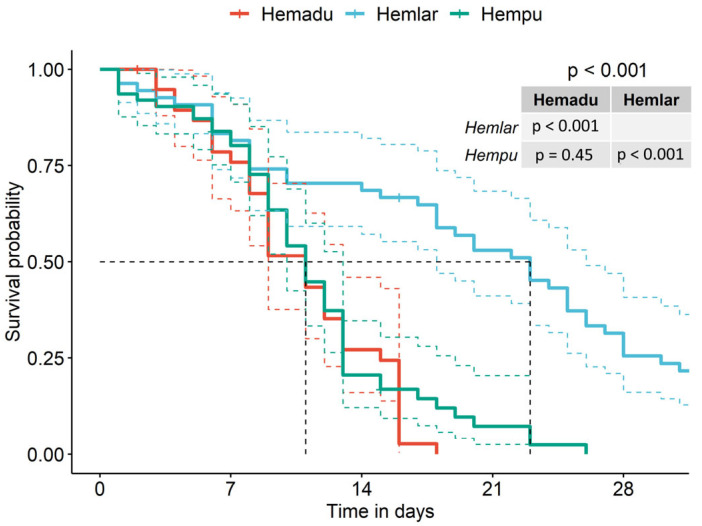
Survival curves and confidence intervals of mites fed on hemolymph of larvae (Hemlar, red curve), pupae (Hempu, light blue curve), or adult bees (Hemadu, green curve). The dashed black line shows the median survival for the three groups. Colored dashed lines surrounding the survival curves represent the 95% confidence interval. *p*-value resulting from the log rank test is shown in the top right corner of the graph along with pairwise *p*-values comparing the different feeding groups.

In our artificial conditions, survival reached more than 70% of still alive mites at the end of the first week (day 7) in each of the three rearing conditions (Figure 2). The parasite was significantly more likely to stay alive for a longer period when fed on larval hemolymph compared to pupal or adult hemolymph (log rank test df = 2, χ^2^ = 42.9, *p* < 0.001). The median survival even reached 23 days in the case of larval hemolymph. Pupal and adult hemolymph solutions do not result in significant survival differences, and the median survival equals 11 days (Figure 2).

### 2.2. Bioassay 2: Feeding on Treated Larval Hemolymph Has No Detrimental Effect on V. destructor Survival

After ensuring that storage at −20 °C did not have any impact on parasites’ survival (Figure S2), larval hemolymph was treated differently and inserted in Parafilm™ dummies to feed female mites (Bioassay 2 in Table 1). More specifically, filtration at 0.2 µm led to a cell-free hemolymph preparation, while heating at 65 °C for 7 min limited melanization [20] and released the cell contents into the medium. Eukaryotic cells exposed to high temperatures (above 55 °C) indeed suffer instant necrosis after membrane rupture [27,28]. Solutions were prepared as described in Table 1 and stored at −20 °C.

**Figure 3:**
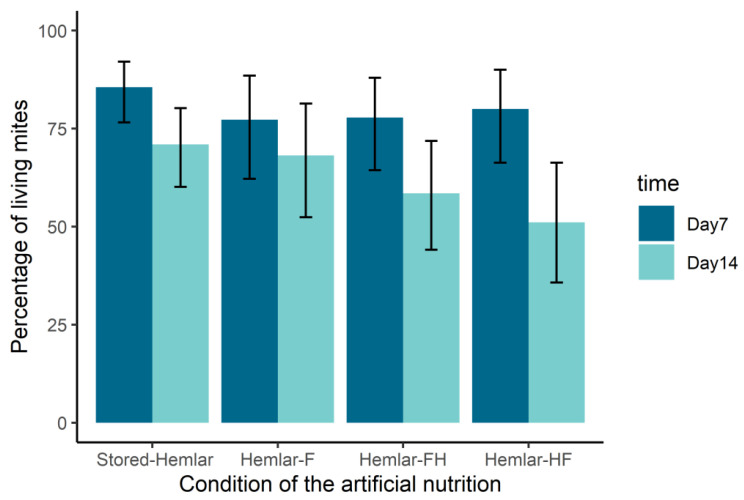
Survival of mites fed on larval hemolymph under different treatments (in % ± CI95). Prior to its insertion in Parafilm™ dummies, hemolymph was heated (Stored-Hemlar), filtered (Hemlar-F), or both heated and filtered (Hemlar-FH for the filtered then heated condition and Hemlar-HF for the heated then filtered condition). No significant difference was highlighted either on day 7 (dark blue) or on day 14 (light blue).

In these conditions, mites survived for seven days in more than 77% of cases regardless of the hemolymph treatment. Although slight differences can be observed in Figure 3, no significance was reached between the four conditions (GLM, df = 3, χ^2^ = 2.06, *p* = 0.56). On day 14, the proportion of alive mites remained above 50%. More precisely, it ranged from 51.1% [CI95: 35.8–66.3] for heated cell-free hemolymph (Hemlar-HF) to 70.9% [CI95: 60.1–80.2] in the case of only heated hemolymph (Stored-Hemlar). Again, no significant difference between the four conditions was noticed at day 14 (GLM df = 3, χ^2^ = 5.98, *p* = 0.11).

### 2.3. Bioassay 3: Artificial Feeding with Synthetic Diet Impacts V. destructor Survival

To mimic hemolymph, PBS solutions supplemented with sugars or sugars and yeast extracts were tested (Table 1). Both glucose and fructose are major hemolymph carbohydrates detected at a concentration of approximately 5 mg/mL each in adult hemolymph, in addition to the glucose disaccharide trehalose (30 mg/mL) [29]. The concentrations used here (50 mg/mL), higher than those observed in natural adult bee hemolymph, were chosen based on preliminary results (Figure S3). In our second feeding condition, yeast extract was added to the carbohydrate solution to simulate amino acid and vitamin uptakes [30]. Again, concentrations of yeast extracts (40 mg/mL) were chosen to be included within a suitable range determined during preliminary experiments (Figure S3). These two test conditions were compared to starved mites, PBS-fed or hemolymph-fed mites.

**Figure 4:**
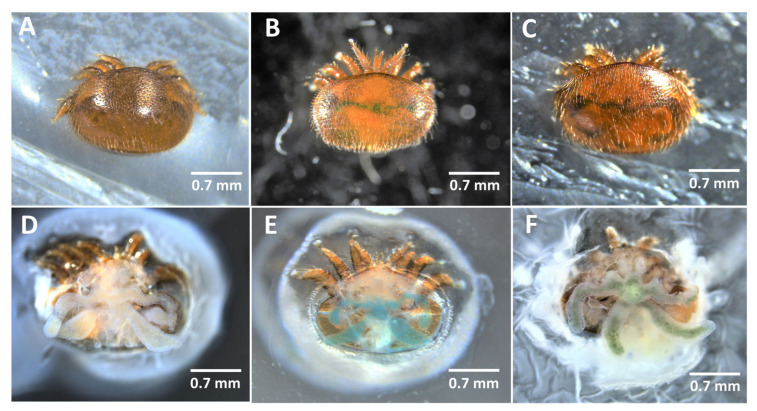
Pictures showing the coloration of *V. destructor* guts through the cuticle (upper panels) or after dissection (lower panels). (**A**,**D**): Mites that feed on a natural uncolored diet; (**B**,**E**): Mites that feed on a synthetic Blue-FCF colored diet; (**C**,**F**): Mite that feed on Blue-FCF colored larval hemolymph.

**Figure 5:**
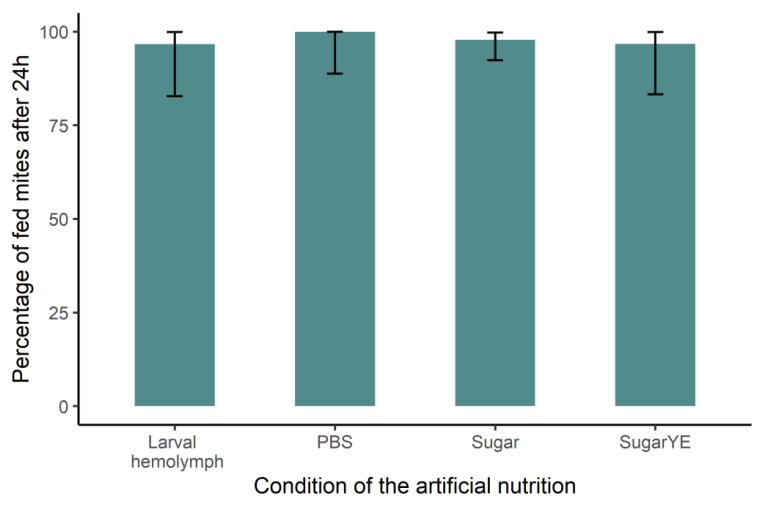
Feeding success of mites fed on synthetic solutions in comparison with mites fed on larval hemolymph (Stored-Hemlar). Synthetic solutions consisted of PBS (control group), PBS supplemented with glucose and fructose (Sugar), or with glucose, fructose, and yeast extract (SugarYE). The coloration of the gut was checked 24 h after the beginning of rearing. No significant difference was detected between the four groups (Larval hemolymph N = 30; PBS control N = 31; Sugar N = 92; SugarYE N = 31).

The first step was to ensure that mites were fed in such artificial conditions. By coloring the feeding media, we managed to verify that after 24 h, the feeding rate was over 95% in every condition (Figure 4 and Figure 5). No significant difference was detected between bee larval hemolymph and sugar-containing PBS (Biased reduced GLM: df = 3, χ^2^ = 0.26, *p* = 0.97).

**Figure 6:**
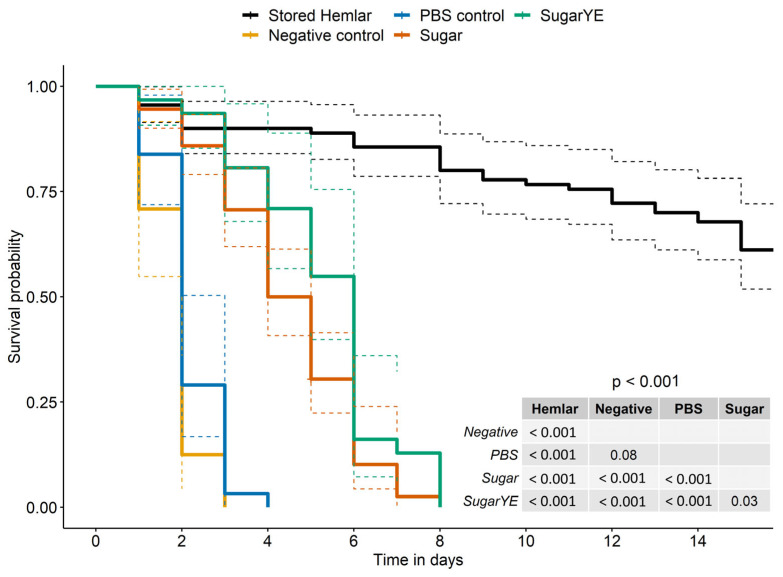
Survival curves and confidence intervals of mites fed synthetic solutions or larval hemolymph. Negative control (yellow curve) = starved mites without feeding dummies; PBS Control (blue curve) = mites with PBS-filled Parafilm™ dummies; Sugar (red curve) = mites fed on a sugar-supplemented PBS solution included in dummies; SugarYE (green curve) = mites fed on sugar and yeast extract supplemented PBS solution included in dummies. Colored dashed lines surrounding the survival curves represent the 95% confidence interval. The *p*-value resulting from the log rank test is shown in the bottom right corner of the graph along with pairwise *p*-values comparing the different feeding groups.

Survival was highly impacted by the type of artificial solution used to feed *V. destructor* (log rank test df = 4, χ^2^ = 257, *p* < 0.001). Mortality was the highest for starved mites (negative control), and a slight tendency to reduce mortality was associated with the presence of PBS-filled dummies. The supplementation with glucose and fructose (Sugar) was sufficient to improve the survival of artificially fed mites compared to sugar-free PBS-fed or starved mites (PBS control and negative control, respectively; Figure 6). The median survival was around five days for mites fed with PBS supplemented with sugar or six days when yeast extract was added to the sugar supplementation (Figure 6; Sugar and SugarYE, respectively). The small increase associated with the addition of yeast extracts was significant. However, whatever the synthetic solution tested, survival was always significantly lower than in the case of hemolymph-fed mites (Figure 6).

### 2.4. Protein Content of Filtered Hemolymph

**Figure 7:**
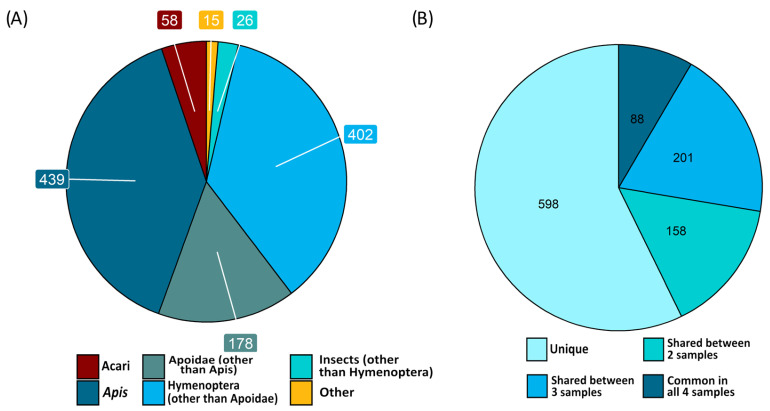
(**A**) Number of proteins detected in filtered larval hemolymph and systematic origin of the matching protein sequence. (**B**) Number of identified proteins that were found only once or shared between two, three, or four hemolymph samples from our three different colonies.

The protein content of filtered larval hemolymph (similar to the Hemlar-F condition in Table 1) was analyzed by off-gel proteomics. Four different larval samples collected from three different colonies were used (Table S1). A total of 1118 proteins were detected, matching mostly insect proteins (93.5%) and, more precisely, Hymenoptera species (91.1%, Figure 7A). The small proportion remaining (6.5%) can be imputed to the presence of *V. destructor* and bee pathogens such as *Nosema* spp. or viruses, with 5.2% of proteins matching Acari species and 1.3% of proteins matching mostly unicellular species. Among the 1045 insect proteins, we focused on a set of 88 proteins that were consistently detected in our samples from three different colonies (Figure 7B). Once uncharacterized proteins and redundant identifications were processed, a shortlist of 54 proteins remained. This set likely consists of the most frequent and abundant proteins naturally present in bee larval hemolymph (Table 2). The 54 proteins could thus be relevant candidate nutrients required for *V. destructor* physiology. Immunity and development are the most represented functions, with 39% of the identified filtered hemolymph proteins being involved in each of these functions. Besides tegument and extracellular matrix molecules, lipid transport and energy metabolism proteins such as Vitellogenin, Apolipophorin, Hexamerin, and Transferrin are also well represented (8 of the 54 proteins; Table 2).

## 3. Discussion

Provided with 3D tube-shaped Parafilm™ dummies filled with nutritional liquids, *V. destructor* females are able to feed under laboratory conditions. Specifically, in flat Petri dishes (Ø 5 cm), the parasite can detect odorless dummies and pierce the Parafilm™ membrane to ingest a liquid diet, whether synthetic or natural. This finding agrees with our previous work showing that under artificial conditions and deprived of olfactive cues, the parasite’s detection of a food source relies on shape-related information [21]. Using Parafilm™ dummies filled with the appropriate feeding solution, we were thus able to maintain mites for weeks. Although rearing methods have already been described [18,19,20,22], the capacity to feed a high number of mites systematically and easily under laboratory conditions is new and promising. Furthermore, the experimental design we developed in this work allowed us to test a variety of diets either completely synthetic or of natural origin.

Larval, pupal, or adult honey bee hemolymph has long been described as the only component of the *V. destructor* diet in natural conditions, until a recent study showed that adult fat body could also be targeted by the mite [22]. In our artificial conditions, the consumption of hemolymph collected from larvae, pupae, or adults was associated with a high survival rate after seven days of rearing. Pure undiluted heated hemolymph is thus sufficient to maintain mites for a week or more. After the first week, the mites survived longer when fed on larval rather than pupal and adult hemolymph. The composition of insect hemolymph varies between developmental stages [101,102,103]. Under our experimental conditions, larval hemolymph could represent an energetically richer food source more suitable to maintain *V. destructor,* whereas pupal and adult hemolymph could be less nutritional. In a natural environment, this lack of nutrients in the adult hemolymph could be compensated for by the consumption of fat body, as described by Ramsey et al. [22]. Since the hemolymph we collected from adult heads is unlikely to contain fat body cells [104], the nutrient deficiency could lead to premature death of *V. destructor* in our experiment. However, our findings do not completely match the work of Ramsey et al. [22], as mites fed on untreated adult hemolymph through a flat Parafilm™ membrane did not survive for more than two days in their study. This divergence could be due to our experimental designs, which differ both in the shape of the Parafilm™ membrane used and in the treatment applied to hemolymph. Indeed, in our study, adult hemolymph was heated for seven minutes at 65 °C to limit early melanization or contamination of the food, which could lead to survival differences in fed mites. This hypothesis, however, seems unlikely, as a survival rate of 80% [CI95: 59.3–93.2] was later observed on day 7 when using untreated adult hemolymph (Table S2). On the other hand, compared to flat Parafilm™ membranes, the 3D rounded shape of the feeding membrane used in our study had an arrestant effect on the mite [21]. This might facilitate the finding of food sources or even stimulate feeding behavior, which could explain the differences with previous studies [22,105].

In any case, the honey bee larva was the most suitable stage for sampling hemolymph as a food source for *V. destructor*. Indeed, in addition to the easy collection of large volumes with only a few individuals, parasites fed on larval hemolymph survived for more than 2 weeks in our study. This longevity is similar to what has been observed when the mite is kept on a natural host under laboratory conditions [106]. This means that female mites obtain the nutrients they need from larval hemolymph, either directly from cell-free hemolymph or from crude hemolymph. We showed that filtered unheated larval hemolymph (i.e., plasma) was as effective as heated hemolymph to feed parasites for weeks. As a result, free-floating hemocytes in plasma appear unnecessary for *V. destructor* nutrition, and plasma should therefore contain the vast majority of nutrients required for mite survival. Studying honey bee hemolymph plasma molecular composition is therefore a priority for understanding the physiological and nutritional needs of *V. destructor*.

Honey bee hemolymph is known to contain a variety of carbohydrates, lipids, free amino acids, and proteins [102,107,108]. Simple carbohydrates such as glucose, fructose, and trehalose are among the most abundant [29,109,110,111]. A mere fructose and glucose solution can thus simulate an extremely simplified version of bee cell-free hemolymph. In our bioassays, the use of such solutions led to a non-negligible survival gain compared with our control PBS solution, reaching levels similar to or higher than those observed with standard complex synthetic solutions [18,19,20]. Glucose and fructose thus represent two of the most important nutrients sought by the parasite. In addition to carbohydrates, other metabolites, such as fatty acids or carboxylic acids, were detected in adult bee hemolymph [107] along with several abundant immune or lipid transport proteins and a variety of free amino acids [101,107,112,113]. In honey bee juvenile stages, studies are scarce and show that the protein content can vary between mobile feeding larvae and immobile fasting and metamorphosing pupae [101,102,114]. In our study, protein supplementation was tested through the addition of yeast extracts, which mostly consist of free amino acids and polypeptides [30]. The addition of yeast extracts to the sugar solution significantly improved the survival of artificially fed mites. Nonetheless, survival rates were far from the results obtained with filtered hemolymph. This highlights that some crucial nutrients remain unknown and hidden in the complexity of the hemolymph, perhaps in the form of whole bee proteins. In *V. destructor*, some proteins obtained from the parasitized bee can indeed act as essential nutrients on their own and not through digestion. Honey bee Vitellogenin has long been known to be found in the parasite’s reproductive organs and hemocoel. More recently, a dozen additional bee proteins were found in the mite after it fed on its host [26]. The amino acid and polypeptides mimicked by the addition of yeast extract thus could not be sufficient for the mite survival, and specific honey bee proteins absent from these artificial feeding solutions could be required by the parasite.

The exact protein content of bee larval cell-free hemolymph was studied in our experiment, and a list of 54 omnipresent proteins was highlighted in each of our samples. Although additional interesting candidate molecules may be missing from this list, these commonly found proteins likely include many of the most frequent and abundant nutrients in bee larval plasma. Unsurprisingly, as hemolymph came from the last larval instar before metamorphosis, the proteins involved in insect development were highly represented [115]. Immune factors were also abundant in our samples, which is understandable, as these proteins are secreted directly into the hemolymph, mostly by hemocytes and fat body tissue [116]. The infectious background of larvae and physical wounds inflicted on the bees during hemolymph collection could also have triggered an immune/inflammatory reaction [114,117]. Nutritionally, the proteins involved in energy metabolism or lipid transport and storage could be particularly interesting. Such proteins might also be present as digested peptides in insect hemolymph, which cannot be discriminated against by our proteomic analyses. In any case, *Varroa destructor* females start their energy-consuming reproductive phase as soon as they enter a fifth instar larva cell, and the first 12 h are crucial for the initiation of reproduction in terms of olfactive signals [118]. The food ingested during the first hours after infestation could also be essential, as it represents the initial energy input used during reproduction. In this case, Vitellogenin, Hexamerin, Apolipophorins, or Transferrins seem to be relevant candidates for the physiological requirements of mites. Recently, Ramsey et al. [26] showed that these honey bee proteins can be directly hijacked by the parasite and accumulate in the females’ body and eggs. The effect of such proteins in artificial feeding media remains to be elucidated. In addition, proteins are not the only nutrients targeted by *V. destructor,* and future metabolomics analyses should shed light on lipids, carbohydrates, or vitamins that may be of particular importance to *V. destructor*.

In conclusion, this study brings us closer to understanding mites’ physiological requirements. From a methodological point of view, the protocol presented here could provide a sustainable procedure for maintaining mites under laboratory conditions, even in the absence of bees. So far, the mean longevity of the mites has reached 3 weeks when fed larval hemolymph, with a maximum of 56 days observed twice. The results obtained with filtered hemolymph also highlight that it is theoretically possible to create a synthetic solution that enables the maintenance of mites for days. The characterization of the nutrients required in such a solution, although fastidious, would be of prime interest for *Varroa* research. Compared with existing artificial feeding protocols for *V. destructor*, which rely on complex synthetic solutions made up of a mixture of cell culture media [18,19,20,21], we described here a simplified sugar solution that achieves similar survival rates, and shed light on several candidate proteins that need to be tested under artificial conditions. This new feeding protocol will be a helpful tool for research about many diverse aspects of *V. destructor* biology and could give rise to new questions about the nutrition of other hematophagous parasites.

It will also allow the development of sustainable rearing techniques without any living hosts, opening a path to new studies under entirely controlled conditions. This could lead to new opportunities in exploring biological control methods against this parasite and reducing its threat to honey bees.

## 4. Materials and Methods

### 4.1. Biological Material

#### 4.1.1. Mites and Bees

Our studies were conducted according to the European ethics laws for scientific research currently in force (Directive 2010/63/EU of the European Parliament and the Council of 22 September 2010 on the protection of animals used for scientific purposes). Eight infested honey bee colonies were maintained on the university campus (Albi, France). *Varroa destructor* adults were collected during spring, summer, and autumn 2022 from sealed brood frames 8 to 12 days postcapping. Only mature females were used as males and juvenile stages are unable to pierce the bee cuticle and depend on their mother to feed. Young and not fully melanized adult females were also excluded.

#### 4.1.2. Hemolymph Collection

Regardless of the honey bee stage, hemolymph collection was carried out on ice to maintain low temperature and avoid rapid melanization and proteolysis. Larval hemolymph was sampled by puncturing larvae with sharp tweezers or entomological pins. A drop of hemolymph was collected using a micropipette and the volume was transferred into a 1.5 mL microcentrifuge tube. About 30 larvae are needed to collect around 1 mL of larval hemolymph. Pupal hemolymph was sampled following a similar process. The abdomen was punctured and the thorax was pressed slightly to collect a droplet of hemolymph with a micropipette. At this stage, the hemolymph is full of suspended fat body cells [26,119], so the collected samples were not as clear and pure as larvae or adults’ hemolymph. Around 30 pupae are necessary to gather 400 µL of pupal hemolymph.

To sample adult hemolymph, freshly freeze-killed worker bees from brood frames were used, and the method described by Borsuk et al., 2017 [104] was followed. Around 160 bees were kept on ice, and their antennae were removed. The bee thorax and abdomen were slightly pressed, and a droplet of hemolymph was collected from the site of the removed antenna using a micropipette. The droplets were transferred into a 1.5 mL microcentrifuge tube kept on ice until a sufficient volume was acquired. Around 160 bees allow the collection of 400 µL of hemolymph.

### 4.2. Artificial Rearing

#### 4.2.1. Hemolymph as a Feeding Solution

During preliminary testing, pure untreated larval hemolymph was fed to the mites. However, due to risks of clotting and contamination, untreated hemolymph can quickly become unsuitable for *V. destructor* feeding after a few days at 34 °C (Figure S1). To avoid contamination and limit clotting or melanization, freshly collected larval, pupal, or adult hemolymph was heated at 65 °C for 7 min (as in [20]) before being included into Parafilm™ dummies. In our first bioassay, we explored the impact that the bee developmental stage used as a source of hemolymph could have on *V. destructor* survival (Table 1).

In a second bioassay, different treatments were applied to larval hemolymph. More specifically, filtration at 0.2 µm and heating at 65 °C for 7 min were conducted in order to obtain cell-free hemolymph with limited risks of melanization and contamination [20]. Heating also released the cell contents into the medium, as eukaryotic cells exposed to high temperature (above 55 °C) suffer instant necrosis after membrane rupture [27,28]. Solutions were prepared as described in Table 1 and stored at −20 °C. We also ensured that cold storage of our nutritive solutions was harmless by comparing the outcome of artificial feeding using freshly collected or frozen hemolymph (Figure S2).

#### 4.2.2. Synthetic Diet

As alternatives to hemolymph, synthetic solutions were fed to mites in a third bioassay (Table 1), and PBS was used as control. In the first feeding condition, a mix of fructose (50 mg/mL) and glucose (50 mg/mL) was added to PBS. These carbohydrates are nutrients naturally present within the hemolymph in the form of mono- or disaccharides. More precisely, in addition to trehalose (30 mg/mL), both glucose and fructose were detected at a concentration of approximately 5 mg/mL in adult hemolymph [29]. The concentrations used here, higher than those observed in natural adult bee hemolymph, were chosen based on preliminary results (Figure S3). In our second feeding condition, yeast extract was added to the carbohydrate solution to simulate proteins and vitamins uptakes [30]. Again, concentrations of yeast extracts (40 mg/mL) were chosen to be included within a suitable range determined during the preliminary experiments (Figure S3). In these two conditions, as well as in the PBS control, feeding solutions were colored using 0.5% artificial Blue-FCF food dye (Vahiné, France) to assess the feeding success of the mites. In all cases, the synthetic feeding solutions were filtered at 0.2 µm to limit the risk of microbial contamination.

#### 4.2.3. Artificial Feeding Chambers and Bioassays

Rearing chambers consisted of Petri dishes (Ø 5 cm) covered with stretched Parafilm™ to limit the effect of static electricity observed on plastic surfaces [120]. The chambers were sterilized under UV light for 20 min. Under a fume hood, two tube-shaped dummies were fashioned from 2 × 2 cm Parafilm™ pieces fully stretched to a thickness of around 16 µm according to Posada-Florez et al. [19]. First, 90 microliters of feeding medium were deposited at the center of the stretched Parafilm™. The Parafilm™ was then carefully folded to create a tube-shaped dummy (Figure 1). In each chamber, 10 to 12 mites from the same colony can be followed at once. Under all conditions, the feeding solutions were renewed twice a week. The survival of mites was recorded daily for every condition through simple observation under a stereomicroscope.

In the first and second bioassays, parasite survival was investigated over 56 and 21 days, respectively. In the case of synthetic diet (Bioassay 3), the survival was also recorded daily, and the feeding success of mites was assessed after 24 h using the dye included in the medium. The blue dye can be seen throughout the parasite’s cuticle, although dissection can be performed to confirm it [121] (Figure 4). An additional sample of 30 mites was also fed on colored larval hemolymph to allow comparison with synthetic diets.

### 4.3. Proteomic Analyses

#### 4.3.1. Sample Processing and Bottom-up Proteomics

The filtered bee larval hemolymph samples (100 µL) were treated with an equal volume of pure hexafluoroisopropanol surfactant (HFIP, Sigma Aldrich, Saint-Quentin-Fallavier, France) and incubated at 4 °C for 4 h while shaking. The HFIP was totally evaporated by centrifugation under vacuum (Labconco, Kansas City, MO, USA), and the hemolymph proteins were reduced, alkylated, and digested as previously described [122,123]. The samples were then stored at −20 °C and dried out prior to proteomics analyses. Lyophilized samples were resuspended in formic acid 0.1% in water. Protein concentration was determined using Pierce 660-nm Protein Assay Reagent (Thermofisher, Rockford, IL, USA). Samples were analyzed on a nanoUPLC system (nanoAcquity, Waters, Milford, MA, USA) coupled to a quadrupole-Orbitrap hybrid mass spectrometer (Q-Exactive plus, Thermo Scientific, San Jose, CA, USA). The system was fully controlled using XCalibur software (v3.0.63; Thermo Fisher Scientific, Waltham, MA, USA). The UPLC system was equipped with a Symmetry C_18_ precolumn (300 Å, 20 × 0.18 mm, 5 µm particle size, Waters, Milford, MA, USA) and an ACQUITY UPLC^®^ BEH C_18_ separation column (130 Å, 75 µm × 200 mm, 1.7 µm particle size, Waters). The solvent system consisted of formic acid 0.1% in water (solvent A) and 0.1% formic acid in acetonitrile (solvent B). Peptides (500 ng for each sample) were first trapped for 3 min at 5 µL/min with 99% A and 1% B. Elution was carried out at 60 °C at a flow rate of 350 nL/min, using a linear gradient from 2 to 35% B in 126 min, then 70% B in 4 min and equilibration was carried out for 14 min at 2% B. To minimize carry-over, a column wash (50% acetonitrile for 20 min) was performed between each sample in addition to a solvent blank.

The Q-Exactive Plus was operated in positive ion mode with the source temperature set to 250 °C and spray voltage to 1.8 kV. Full-scan MS spectra (300–1800 *m/z*) were acquired at a resolution of 140,000 at *m/z* 200, with a maximum injection time of 50 ms and an AGC target value of 3 × 10^6^ charges. The lock-mass option was enabled (polysiloxane, 445.12002 *m/z*). Up to 10 most intense peptides (at least doubly charged) per full scan were isolated using a 2 *m/z* window and they were fragmented using higher energy collisional dissociation (normalized collision energy of 27 eV, and dynamic exclusion of already fragmented precursors set to 60 s). MS/MS spectra (200–2000 *m/z*) were acquired at a resolution of 17,500 at *m/z* 200, with a maximum injection time of 100 ms and an automatic gain control (AGC) target value of 1 × 10^5^. The peptide match selection option was turned on. Peak intensities and retention times of reference peptides were monitored in daily.

#### 4.3.2. Proteomics Data Processing

Proteome Discoverer 2.5 (Thermo Fisher Scientific, Waltham, MA, USA) was used for bottom-up proteomics sequencing and to quantify the proteins based on the intensities of the precursor ions of unique and razor peptides. A processing workflow and a consensus workflow were used to characterize and validate/quantify the proteins in each analyzed sample, respectively. A Fasta file protein database containing entries from NCBI gathered 1,532,988 sequences of Hymenoptera, 20,214 protein sequences from *Aethina tumida*, 14,703 sequences from *Tropilaelaps* species, 59,574 sequences from *Varroa* species, 7578 entries from bee viruses, 32,221 entries from *Crithidia* and *Lotmaria* species, and 115,707 entries from mites. The trypsin enzyme, a precursor mass tolerance of 20 ppm, a fragment mass tolerance of 0.5 Da, and the chemical modifications (Oxidation/+15.995 Da as dynamic on methionine and tryptophan residues and pyridylethyl/+105.058 Da as static on cysteine) were selected for subsequent data processing. The datasets were aligned to extract for each individual file, the LC-MS/MS mapping features. The settings were as follows: A maximum retention time (RT) shift of 10 min., a mass tolerance of 10 ppm, and coarse parameter tuning were set. Protein annotations were successfully validated when the q-value scores were calculated below the false discovery rate (0.05). 

### 4.4. Statistical Analyses

Results were analyzed using R.4.0.4 [124] and graphs were generated using the ggplot2 package [125]. When mortality on day 7 and 14 was compared, binary data (survival 0 or 1) were analyzed using generalized linear models (GLM) with binomial distribution. The feeding success after 24 h was analyzed following the same process.

Survival curves were fitted to the data to follow mortality over time. Data were analyzed using the survfit package in R and log rank tests. If significance was reached, pairwise comparisons with Benjamini-Hochberg corrections were further computed.

## Figures and Tables

**Table 1:** Details of the conditions tested in three different bioassays. Bee developmental stage and hemolymph treatment (heating, filtration, storage) are shown in the table, along with abbreviations used in the graphs and sample size N. In rare cases, there is a slight difference between the initial sample size (N_0_) and the sample sizes after one or two weeks because some mites escaped from their chambers in the course of our experiment (Figure S2).

Bioassay—Factor Tested	Condition	Treatment Applied to Feeding Solution			Abbreviation	Number of Mites Tested (N)
		Heated (65 °C, 7 min)	Filtered (0.2 µm)	Storage (−20 °C)		
1—Origin of hemolymph	Larva	Yes	No	No	Hemlar	64
	Pupa	Yes	No	No	HemPu	62
	Adult	Yes	No	No	HemAdu	39
2—Treatment of larval hemolymph	Stored	Yes	No	Yes	Stored-Hemlar	90
	Filtered	No	Yes	Yes	Hemlar-F	44
	Heated then filtered	Yes (firstly)	Yes (secondly)	Yes	Hemlar-HF	57
	Filtered then heated	Yes (secondly)	Yes (firstly)	Yes	Hemlar-FH	54
3—Synthetic diet	Negative	No	No	No	Negative control	24
	PBS	No	No	No	PBS control	31
	PBS + Sugar (100 mg/mL)	No	Yes	No	Sugar	92
	PBS + Sugar (100 mg/mL) + Yeast extract (40 mg/mL)	No	Yes	No	SugarYE	31

**Table 2:** List of proteins detected in each of the four filtered larval hemolymph samples from three different colonies. The protein name and the species from the matching protein sequence are indicated, along with the consistency of detection and the probable function described in the literature.

Protein Name	Species	Detected in All or Part of Technical Replicates	Function	Reference
A-agglutinin anchorage subunit isoform X1	*Apis mellifera*	All	Cell-cell adhesion; immunity	Rowley and Ratcliffe (1980) [31]
Abaecin	*Apis cerana*	11/12	Immunity	Casteels et al., (1990) [32]; Plua and sokol (2020) [33]
AF4/FMR2 family member 4	*Apis florea/Nomia melanderi*	7/12	Cell growth/identity; development	Wittwer et al., (2001) [34]
Apidaecin	*Apis cerana*	All	Immunity	Plua and sokol (2020) [33]
Apolipophorins	*Apis mellifera*	All	Lipid metabolism; energy storage; immunity	Cabri et al., (2018) [35]; Kim and Jin (2014) [36]
Apolipoprotein D	*Apis mellifera/Apis cerana*	All	Lipid metabolism; energy storage	Chan and Foster (2008) [37]
Beta-galactosidase	*Apis mellifera*	All	Carbohydrates metabolism	Peng (1980) [38]; Ricigliano et al., (2017) [39]
Bifunctional methylenetetrahydrofolate dehydrogenase/cyclohydrolase, mitochondrial	*Bombus vancouverensis*	8/12	Oxidative stress; development; aging	Tremblay et al., (1995) [40]; Yu et al., (2015) [41]
BMP and activin membrane-bound inhibitor	*Apis cerana*	All	Development	Upadhyay et al., (2017) [42]
chitinase-like protein EN03 isoform X1	*Apis mellifera*	All	Development	Li et al., (2010) [43]
Chymotrypsin inhibitor	*Apis mellifera*	All	Coagulation/immunity	Kim et al., (2013) [44]; Corral-Rodriguez et al., (2009) [45]; Rhoads et al., (2000) [46]
Class E basic helix-loop-helix protein 22	*Apis mellifera*	10/12	Development (transcription factor)	Ledent and Vervoort (2001) [47]; Wan et al., (2016) [48]; Wang et al., (2008) [49]
Collagen alpha-1(IV) chain	*Apis mellifera*	All	Extracellular matrix; development	Pastor-Pareja et al., (2011) [50]; Sutherland et al., (2013) [51]
Cubilin	*Apis cerana*	11/12	Endocytosis	Zhang et al., (2013) [52]
Cuticular protein 2 precursor	*Apis mellifera*	All	Tegument protein; development (preparation of ecdysis)	Hopkins et al., (2000) [53]; Soares et al., (2007) [54]
Endocuticle structural glycoprotein SgAbd-1	*Pseudomyrmex gracilis*	11/12	Tegument protein	Micas et al., (2016) [55]
FABP-like protein	*Apis cerana*	All	Lipid transport/uptake; immunity; development	Caccia et al., (2012) [56]; Chen et al., (2022) [57]; Cheng et al., (2013) [58]
Fibrillin-2	*Apis mellifera*	10/12	Extracellular matrix	Piha-Gossack et al., (2012) [59]
Fibroin heavy chain isoform X1	*Apis mellifera*	9/12	Silk protein	Sutherland et al., (2006) [60]
Flexible cuticle protein 12-like isoform X1	*Apis florea*	All	Tegument protein; development	Rebers et al., (1988) [61]
Floculation protein FLO11 isoform X1	*Apis mellifera*	All	Extracellular matrix; development	Zhao et al., (2020) [62]
Glucose dehydrogenase [FAD, quinone]	*Apis mellifera*	All	Development; immunity	Cox-Foster et al., (1990, 1994) [63,64]
Glycine-rich cell wall structural protein 1 isoform X1	*Apis mellifera*	All	Cuticle protein; development; response to stress	Zhang et al., (2008) [65]; Zhong et al., (2005) [66]
Hexamerin	*Apis mellifera*	All	Storage protein	Martin et al., (2010) [67]
Inactive serine protease scarface	*Apis mellifera*	11/12	Development	Contreras et al., (2021) [68]
Larval-specific very high density lipoprotein precursor	*Apis mellifera*	All	Lipid transport/storage	Shipman et al., (1987) [69]
Leukocyte elastase inhibitor	*Apis mellifera*	All	Coagulation/immunity	Kim et al., (2013) [44]; Corral-Rodriguez et al., (2009) [45]; Rhoads et al., (2000) [46]
Interferon-related developmental regulator 1-like	*Apis mellifera*	All	Development; immunity	Arockiaraj et al., (2014) [70]; Hoffmann et al., (1996) [71]; Stanifer et al., (2019) [72]
Lysozyme	*Apis mellifera*	All	Immunity	Al-Ghamdi et al., (2021) [73]
Neurofilament heavy polypeptide	*Apis mellifera*	All	Neural cytoskeleton; neural development	Bezabih et al., (2017) [74]; Petzold (2005) [75]
OBP13	*Apis mellifera*	All	Olfaction; transport protein in larvae	Forêt and Maleszka (2006) [76]
Odorant binding protein 14 precursor	*Apis mellifera*	All	Olfaction; transport protein in larvae	Forêt and Maleszka (2006) [76]
Omega-conotoxin-like protein 1	*Apis mellifera*	All	Immunity/melanization	Bloch et Cohen (2014) [77]
Peptidyl-prolyl cis-trans isomerase B precursor	*Apis mellifera*	All	Protein folding; development, cell differentiation; oxidative stress and immunity	Wang and Heitmann (2005) [78]; Yoon et al., (2022) [79]
Peritrophin-1	*Apis mellifera*	All	Tegument protein (of the peritrophic membrane)	Park et al., (2016) [80]
Phenoloxidase subunit A3	*Apis mellifera*	All	Melanization/immunity	Wilson-Rich et al., (2008) [81]
Phenoloxidase-activating factor 2 isoform X1	*Apis mellifera*	All	Melanization/immunity	Wilson-Rich et al., (2008) [81]
Plexin domain-containing protein 2 isoform X1	*Apis florea*	All	Clotting; development; immunity	Miller-Delaney et al., (2011) [82]; Thibord et al., (2019) [83]
Odorant receptor 43a-like isoform X1	*Vollenhovia emeryi*	5/12	Olfaction	Liu et al., (2020) [84]
Prisilkin-39 isoform X1	*Apis cerana*	8/12	Silk matrix protein	Jung et al., (2021) [85]
Probable G-protein coupled receptor Mth-like 10	*Pseudomyrmex gracilis*	6/12	Oxidative stress and longevity	Liu et al., (2021) [86]
Protein D2	*Apis mellifera*	All	Brain development	Jørgensen (1983) [87]
Protein mesh isoform X1	*Apis mellifera*	10/12	Cell-cell adhesion; development	Jonusaite et al., (2020) [88]
Putative acyl-CoA-binding protein	*Apis cerana*	All	Lipid metabolism	Majerowicz et al., (2016) [89]
Putative cyclin-dependent serine/threonine-protein kinase	*Apis mellifera*	All	Regulation of transcription; cell division	Zhao et al., (2018) [90]
Secapin-2 precursor	*Apis mellifera*	11/12	Immunity; venom	Al-Naggar et al., (2023) [91]; Doublet et al., (2017)
Secapin-3 precursor	*Apis mellifera*	All	Immunity; venom	Al-Naggar et al., (2023) [91]; Doublet et al., (2017) [92]
Serine protease inhibitor 3	*Apis mellifera*	All	Immunity	Shakeel et al., (2019) [93]
Thymosin beta-a	*Apis cerana*	11/12	Immunity; development	Zhang et al., (2011) [94]
Titin homolog, partial	*Bombus terrestris*	10/12	Muscle protein	Hooper and Thuma (2005) [95]
Transcriptional regulator ATRX homolog	*Apis mellifera*	All	Gene regulation; cell division	Lopez-Falcon et al., (2014) [96]
Transferrin	*Apis mellifera*	All	Iron transport; immunity; energy metabolism	Geiser et al., (2012) [97]; Rodriguez Garcia et al., (2021) [98]
Venom carbohydrate-rich protein precursor	*Apis mellifera*	10/12	Venom component allergen	Peiren et al., (2006) [99]
Vitellogenin-6-like	*Apis dorsata*	All	Immunity; energy metabolism; lipid transport; development	Leipart et al., (2022a,b) [100]

## Data Availability

All data are available upon request. The mass spectrometry proteomics data have been deposited to the ProteomeXchange Consortium (http://proteomecentral.proteomexchange.org, accessed on 10 July 2023) via the PRIDE [126] partner repository with the dataset identifier PXD043487.

## Supplementary Materials

The following supporting information can be downloaded at: https://www.mdpi.com/article/10.3390/ijms241512443/s1.
