# Supplementary material for: Honey Bee Larval Hemolymph as a Source of Key Nutrients and Proteins Offers a Promising Medium for Varroa destructor Artificial Rearing
Source: Int J Mol Sci. 2023 Aug 4;24(15):12443. doi: 10.3390/ijms241512443 (PMC10419257; doi:10.3390/ijms241512443)
Supplement: Supplementary file 1 [file ijms-24-12443-s001.zip › ijms-2528556-supplementary.pdf]

Supplementary material

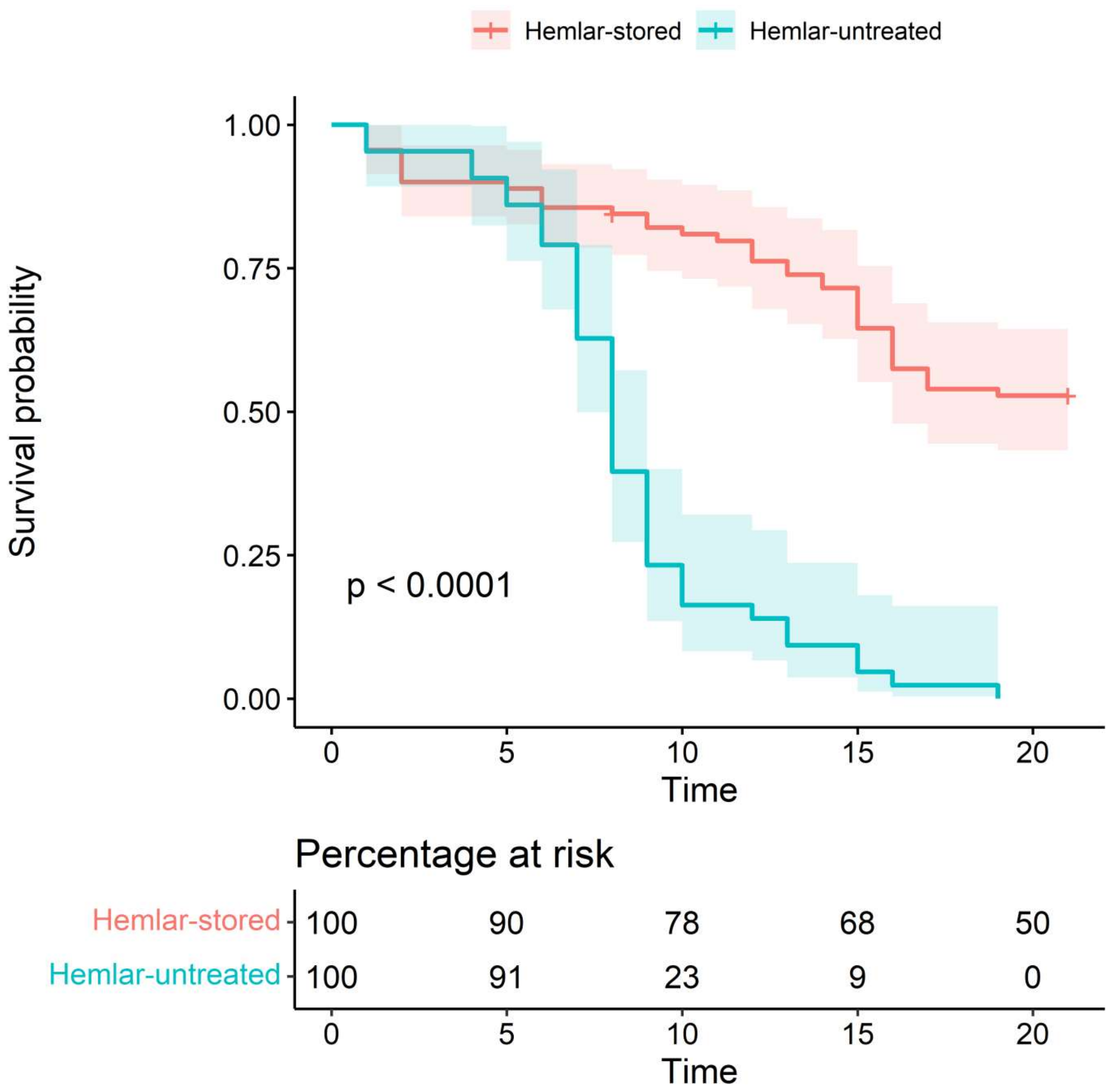

Figure S1. Survival curves highlighting the benefit of hemolymph heating on mites survival. Compared to heated larval hemolymph (N=90), untreated hemolymph (N=33) leads to a significant increase of mortality after the first week (Log Rank test df=1, χ^2=64.8, $p<0.001$). Although it was not directly tested, the mortality may be related to the contamination of untreated hemolymph after a few days at 34°C.

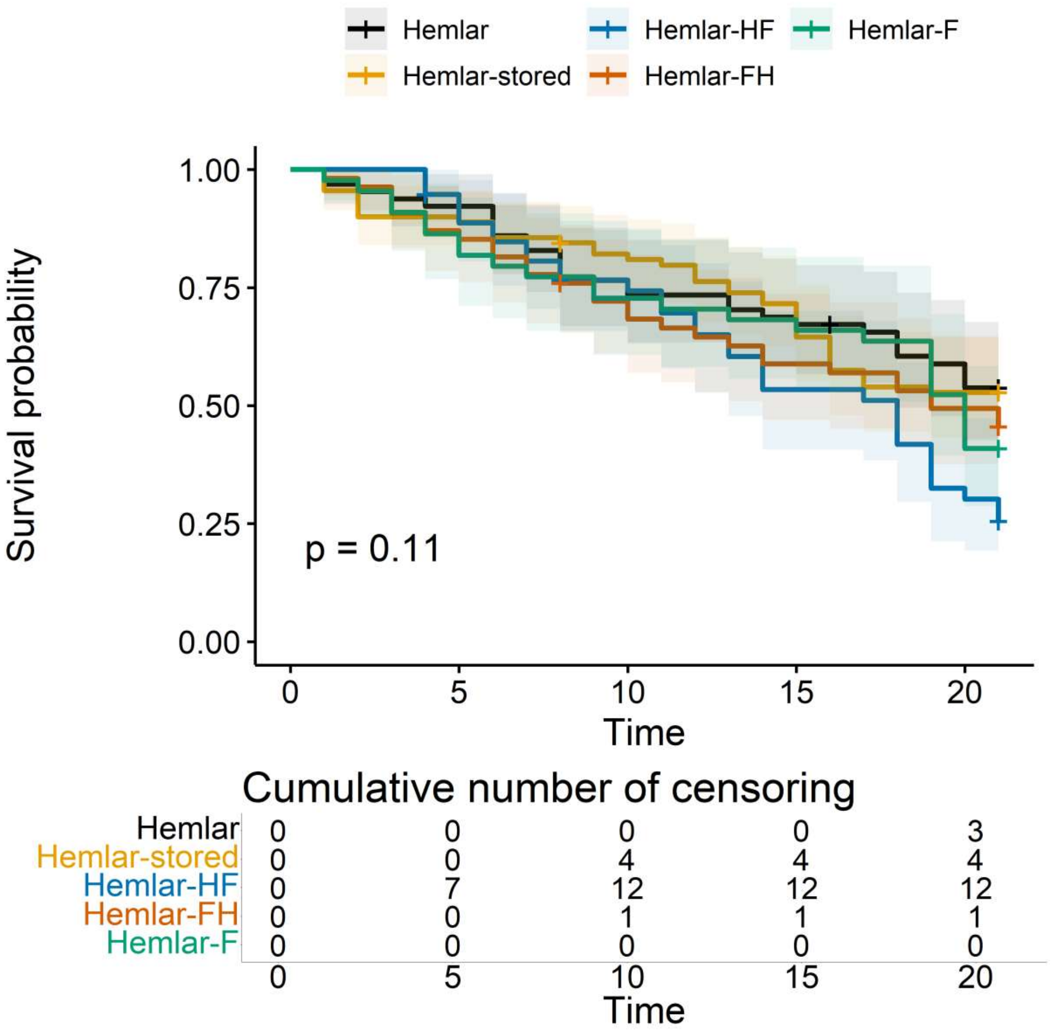

Figure S2 Survival curves of mites fed with differently treated larval hemolymph over the course of the 21 days. No significant difference was detected (Log Rank test df=4, χ^2=7.5 p=0.1). The table shows the cumulative number of censoring due to mites that managed to escape from feeding chambers.

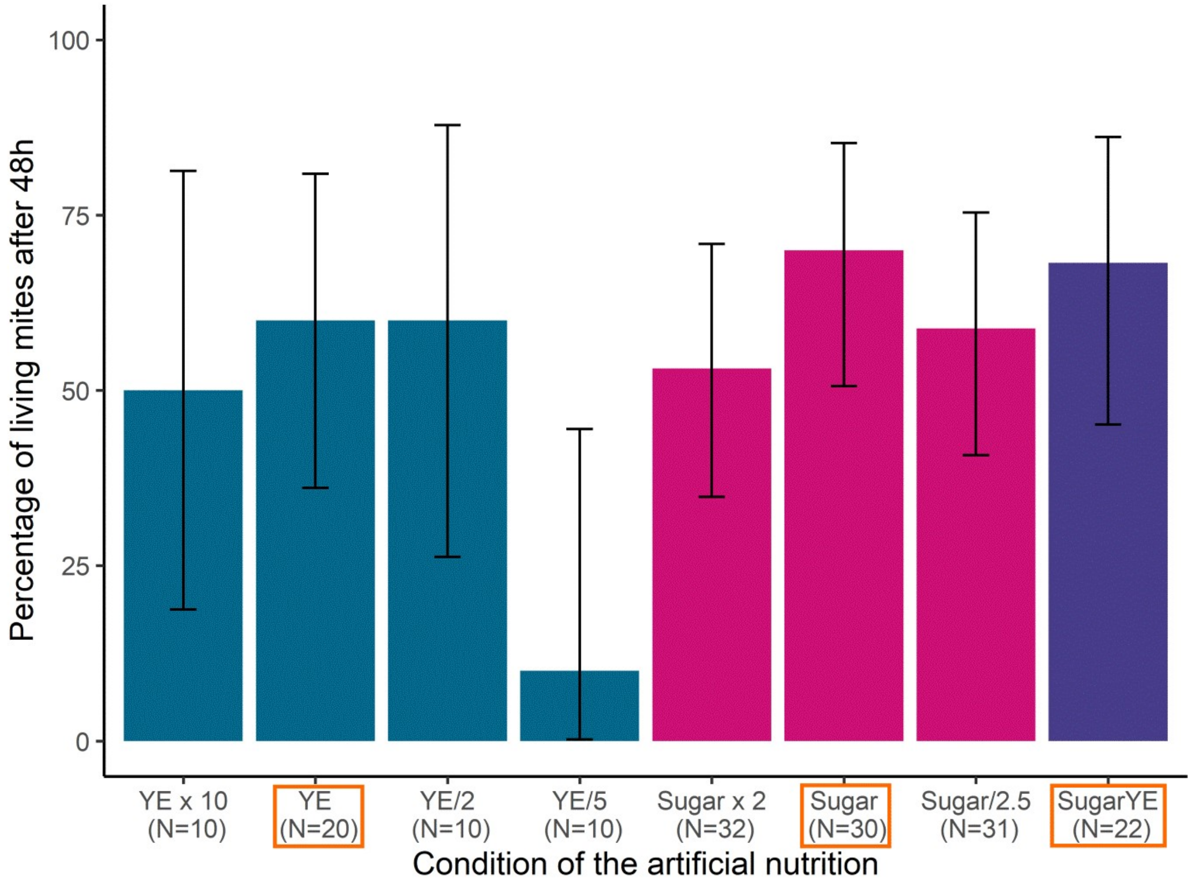

Figure S3. Overview of preliminary results from an artificial rearing of *V. destructor* females using synthetic solutions. Mites were followed over two days in gelatine capsules while they fed on yeast extracts (Blue), sugars (Pink) or on a mix of both (Purple). Concentrations used in the present study are surrounded on the x axis (40mg/ml of yeast extracts and 50 mg/ml of both glucose and fructose). The other conditions are expressed as a relation to this basis concentration.

Table S2. Survival of mites fed with untreated adult hemolymph through our artificial feeding protocol. Adult hemolymph was renewed daily to avoid contamination of the feeding solution.

	**Time in days**			
	D2	D7	D9	D14
Survival of mites (1=alive; 0= dead)	1	1	1	1
	1	1	1	0
	1	1	0	0
	1	0	0	0
	1	0	0	0
	1	0	0	0
	1	1	1	1
	1	1	1	1
	1	1	1	0
	1	1	1	0
	1	1	1	0
	1	1	1	0
	1	1	1	0
	1	1	1	0
	1	0	0	0

	1	1	0	0
	1	1	0	0
	1	1	0	0
	1	1	0	0
	1	1	0	0
	1	1	1	0
	1	1	1	0
	1	1	1	0
	1	1	1	0
	1	0	0	0
**Total percentage of mites alive**	**100**	**80**	**54**	**12**
